# Hami Melon Fruit Quality Variation Across Regions and Its Correlation with Growing Environments

**DOI:** 10.3390/foods15203581

**Published:** 2026-10-09

**Authors:** Kaili Ren, Taoxia Tang, Weiping Kong, Yongquan Su, Huasheng Zhang, Hong Cheng

**Affiliations:** Institute of Vegetables, Gansu Academy of Agricultural Sciences, Lanzhou 730070, China; renkaili66@126.com (K.R.); tangtaoxia@126.com (T.T.); wpk33@126.com (W.K.); gssyq@sohu.com (Y.S.); zhanghuasheng@gsagr.ac.cn (H.Z.)

**Keywords:** *Cucumis melo*, geographical origin, fruit texture, sugar–acid balance, multivariate analysis, planting environment

## Abstract

Fruit quality in Hami melon varies among production sites, but its associations with growing conditions remain insufficiently characterized. This study compared 31 fruit quality traits of ‘Xizhoumi 17’ collected from eight production sites in China in 2025 and explored their relationships with regional climatic conditions and soil properties. Analysis of variance, principal component analysis (PCA), cluster analysis, regression analysis and correlation analysis were used to characterize site-level variation. Fruit quality differed markedly across sites: total sugar content ranged from 4.94 to 13.19 g/100 g·FW, the sugar–acid ratio from 7.99 to 26.28, and texture profile TPA hardness from 24.09 to 43.62 N. Five principal components explained 94.74% of the total variance, and ranked Dachanghe, Xiquan and Huaxi as the three highest-scoring sites. Stepwise regression analysis identified fructose content, TPA hardness, and total sugar content as the core determinants of Hami melon quality, indicating that these traits can serve as fundamental evaluation criteria. Correlation analysis further suggested that adequate light exposure, large diurnal temperature variation, and relatively low air humidity are the three key climatic factors required for producing high-quality Hami melons. In addition, appropriately increasing nitrogen and organic fertilizer application while reducing excessive phosphate fertilizer input could further improve fruit quality. This study establishes a theoretical basis for the high-quality cultivation of Hami melons.

## 1. Introduction

Melon (*Cucumis melo* L.), an annual trailing herb belonging to the genus Cucumis in the Cucurbitaceae family, is cultivated globally, and its harvested area has maintained a sustained upward trend. According to the latest statistics from the Food and Agriculture Organization of the United Nations (FAOSTAT), the global melon cultivation area reached 1,423,348 hectares by 2024, with a total production of 41,512,716.71 tons, representing year-on-year increases of 30.30% and 40.52%, respectively. As a distinctive thick-rinded melon ecotype indigenous to Xinjiang, Northwest China, Hami melon (*Cucumis melo* L. var. *reticulatus* Naud.) is widely acclaimed as the ‘King of Melons’ [1,2]. It is celebrated for its exceptional sweetness, refreshing juiciness, distinct layered flavor, and abundant bioactive functional nutrients—traits that collectively endow this cultivar with outstanding edible and nutritional value [3,4,5,6,7]. Boasting a relatively short growth cycle and considerable economic returns, Hami melon has emerged as a high-value commercial crop that plays an irreplaceable role in boosting the income of local farming communities, and its cultivation has expanded widely to multiple suitable agro-ecological regions across China in recent years [8].

Existing research has made considerable advances in the evaluation of melon quality. A comprehensive texture assessment of thin-skinned melons has been completed systematically [9], while separate investigations focused on thick-skinned varieties identified chewiness, adhesiveness, and resilience as the core indicators for texture evaluation [10]. To screen high-quality melon germplasm resources, in-depth evaluations covering multiple fruit traits of diverse melon materials have been extensively carried out [11,12,13,14]. Furthermore, a study analyzing 18 quality indicators from 32 ‘Yangjiaocui’ melon samples pinpointed fruit vertical diameter, flesh thickness ratio, hardness, total sugar and titratable acid as the most representative quality evaluation indexes for this specific cultivar [15]. Another systematic assessment covering 17 quality indicators across eight different melon varieties concluded that soluble solids, vitamin C, total phenols, ABTS free radical scavenging rate and core sugar–acid-related indicators constitute the essential basis for evaluating the nutritional value of melon fruits [16]. Despite these fruitful achievements, most existing studies have focused predominantly on the genotypic regulation of fruit traits, while the environmental modulation of melon quality across different habitats remains poorly understood. Although limited work has confirmed that growing conditions significantly alter melon fruit quality [17], systematic quantitative analyses linking site-specific environmental conditions to multidimensional fruit quality properties are still largely absent.

Given that habitat-dependent variation in Hami melon fruit quality remains largely unclear, this study used Xizhoumi No. 17 samples collected from eight experimental sites across four provinces and autonomous regions to address four interlinked research objectives: (i) to quantitatively characterize regional variations in fruit morphological traits, textural properties, sugar–acid composition, mineral elements, and antioxidant-related attributes; (ii) to establish an exploratory multivariate ranking system for site-specific fruit quality performance; (iii) to screen out a compact set of candidate indicators applicable to rapid quality assessment and screening; and (iv) to clarify the quantitative associations between fruit quality traits, climatic conditions, and the in situ soil properties of each site. We hypothesized that environments with higher solar radiation and a larger diurnal temperature range would significantly facilitate sugar accumulation in fruit, and that the inherent soil nutrient status of each site would further contribute to compositional quality variation across different habitats.

## 2. Materials and Methods

### 2.1. Study Sites and Plant Material

Fruit and rhizosphere soil were collected from eight commercial Hami melon production sites (Table 1). The cultivar ‘Xizhoumi No. 17’ was used at every site, allowing regional variation to be examined without a deliberate change in cultivar. Site names are used throughout the manuscript as the sampling-unit identifiers.

### 2.2. Climatic Data

To characterize the climatic conditions of each production region, sunshine duration, solar radiation, rainy days, precipitation, evaporation, and relative humidity were obtained from the Geographic Remote Sensing Ecological Network Platform (https://www.gisrs.cn/?data_89/) (accessed on 24 September 2026). Details of the five national standard ground meteorological stations are as follows: Yumen 52,436, 40.2667° N/97.0333° E; Guazhou 52,424, 40.5333° N/95.7833° E; Sanya 59,948, 18.2167° N/109.5833° E; Turpan 51,573, 42.9500° N/89.2333° E; Bayannaoer 53,513, 40.7333° N/107.3667° E. The straight-line distances from each station to the corresponding regional fruit sampling site were 40–70 km (Yumen), ~18 km (Guazhou), ~16.1 km (Sanya), ~83 km (Turpan), and ~77.7 km (Bayannaoer), respectively.

Furthermore, daily maximum and minimum temperatures were sourced from Weather+ (https://lishi.tianqi.com/) (accessed on 24 September 2026), while mean temperature data was obtained from Windy Weather (https://www.qweather.com/) (accessed on 24 September 2026). For each region, records were taken from April to July 2025. Diurnal temperature range was calculated as the difference between the mean daily maximum temperature and the mean daily minimum temperature. All four Yumen sites used the same city-level climatic record.

### 2.3. Soil Sampling and Analysis

#### 2.3.1. Soil Sampling

At each site, soil was sampled from the 0–30 cm layer using a five-point design. Visible stones and plant debris were removed, and equal portions from the five points were homogenized to form one composite sample. Three independently collected composite samples (approximately 500 g each) represented each production site. The samples were placed in labeled polyethylene bags and transported for analysis.

#### 2.3.2. Soil Chemical Analyses

The measured soil variables were pH, organic matter, available phosphorus, available potassium, total nitrogen and cation exchange capacity (CEC).

Soil pH was measured potentiometrically in a 1:2.5 soil-to-water suspension [18]. Available phosphorus was determined via the standard Olsen’s extraction method [18], while available potassium was extracted and measured using the neutral normal ammonium acetate approach [18,19]. Organic matter was measured by potassium dichromate–sulfuric acid oxidation with external heating [20]. Total nitrogen was quantified by the Kjeldahl method, and CEC by ammonium acetate extraction [21].

### 2.4. Fruit Sampling and Quality Measurements

All samples were collected in 2025. Experimental Hami melon plants (cv. Xizhoumi 17) were grown in a plastic tunnel using a standardized single-vine, single-fruit trellis system, with 50 cm plant spacing and 70 cm row spacing. To minimize confounding effects from non-experimental factors, all agronomic practices across the eight sites—including soil preparation, fertilization regimes, routine crop management, and water and nutrient supply—were maintained as consistently as possible to ensure the comparability of data from different locations.

Based on the growth cycle of cv. Xizhoumi 17 and local cultivation experience, 15 fruits of uniform moderate maturity and free from visible pest and disease damage were selected from the experimental plots at each site. These fruits were then randomly assigned to three biological replicates, with five fruits per replicate. The quality evaluation covered five domains: morphological traits, texture profile attributes, sugar–acid composition, mineral elements, and bioactive or antioxidant-related traits.

#### 2.4.1. Morphological Traits

Fruit mass was measured with an electronic balance. Fruit length, equatorial width and flesh thickness were measured using a ruler [12,16]. Fruit shape index was calculated as fruit length divided by fruit width.

#### 2.4.2. Texture Profile Analysis

Flesh texture was characterized using a TMS-Pro texture analyzer (serial no. 19-0210-11). Texture profile analysis generated TPA hardness, adhesiveness, cohesiveness, springiness, gumminess and chewiness. Sample preparation and instrument settings followed Yang et al. [10].

#### 2.4.3. Soluble Sugars and Organic Acids

Fructose, glucose, sucrose, malic acid and citric acid were quantified via high-performance liquid chromatography [22]. Total sugar was calculated as the sum of glucose, fructose and sucrose, and total acid as the sum of citric acid and malic acid. The sugar–acid ratio was then derived by dividing total sugar content by total acid content. Finally, the fruit sweetness value was calculated using the following formula: Fruit sweetness value = 1.75 × fructose + 0.7 × glucose + 1.0 × sucrose [13].

#### 2.4.4. Mineral Elements

Calcium, magnesium, iron, zinc and potassium were quantified by inductively coupled plasma-mass spectrometry according to Zhou et al. [23].

#### 2.4.5. Bioactive Compounds and Antioxidant Capacity

Total polyphenol content was determined by the Folin–Ciocalteu assay using gallic acid as the calibration standard and measuring absorbance at 765 nm [24]. Total flavonoid content was determined by the NaNO_2_-Al(NO_3_)_3_-NaOH colorimetric method using rutin as the standard and measuring absorbance at 500 nm [24].

DPPH and ABTS radical scavenging capacities were measured as described by Shao et al. [16]. Protein content was determined by the automated Kjeldahl method, and ascorbic acid was determined by high-performance liquid chromatography [25].

### 2.5. Statistical Analysis

Data are presented as mean ± standard deviation (SD) of three biological replicates. Site effects on individual fruit and soil variables were assessed by one-way analysis of variance in SPSS 26.0; post hoc mean separation was interpreted at *p* < 0.05. The coefficient of variation (CV) of each fruit quality index was defined as the ratio of its corresponding standard deviation to its mean value.

For multivariate analyses, all repeated measurements of each trait at each site were standardized prior to PCA. Following the criterion of eigenvalues greater than 1, five principal components were ultimately extracted. The weight for each selected principal component was determined by the proportion of its variance contribution. For each sample, the scores of the first five principal components were then weighted by their corresponding weights to generate the comprehensive quality score (F), which characterized the overall fruit quality of Hami melons from diverse locations.

Additionally, cluster analysis (Euclidean distance, between-groups linkage) and stepwise regression analysis were performed using SPSS 26.0, and the corresponding correlation heat maps (Pearson method) were generated in Origin 2024.

## 3. Results and Analysis

### 3.1. Environmental Analysis of the Hami Melon Production Regions in China

#### 3.1.1. Climatic Analysis

The five production regions exhibited a pronounced climatic gradient between April and July (Table 2). Among them, Yumen and Guazhou had the highest mean monthly sunshine duration and solar radiation during the Hami melon growing season, while Sanya registered the lowest values. The mean diurnal temperature range was between 5.25 and 11.50 °C, peaking at Yumen, Guazhou and Bayannaoer, and reaching the minimum at Sanya. Moreover, mean monthly relative humidity, precipitation, and rainy days all peaked in Sanya (94.42%, 166.58 mm, 13.00 d, respectively), and were lowest in Turpan (19.17%, 0.03 mm, 0.25 d, respectively).

#### 3.1.2. Soil Chemical Properties

Soil properties also differed among sites (Table 3). All soils were neutral to moderately alkaline (pH 7.50–8.39). Organic matter ranged from 9.17 ± 0.16 to 19.70 ± 0.25 g/kg, available phosphorus from 7.84 ± 0.10 to 50.60 ± 0.20 mg/kg, available potassium from 132.33 ± 0.67 to 414.67 ± 1.45 mg/kg, total nitrogen from 0.38 ± 0.01 to 1.00 ± 0.01 g/kg and CEC from 2.07 ± 0.07 to 5.93 ± 0.09 cmol(+)/kg. Dachanghe had the highest organic matter and available potassium, whereas Lianfeng had the highest total nitrogen and CEC.

**Table 3:** Analysis of soil nutrition indexes.

Site	pH	Organic Matter (g/kg)	Available P (mg/kg)	Available K (mg/kg)	Total N (g/kg)	CEC (cmol(+)/kg)
Xiquan	7.95	16.73 ± 0.13 b	22.43 ± 0.43 d	167.67 ± 0.33 f	0.81 ± 0.02 c	3.67 ± 0.03 c
Dachanghe	7.98	19.70 ± 0.25 a	47.53 ± 0.71 b	414.67 ± 1.45 a	0.89 ± 0.01 b	3.13 ± 0.03 d
Huaxi	8.20	9.90 ± 0.08 ef	7.84 ± 0.10 h	230.00 ± 1.53 c	0.45 ± 0.01 f	2.23 ± 0.09 f
Yuanquan	7.95	9.17 ± 0.16 f	18.20 ± 0.15 f	212.67 ± 0.88 e	0.38 ± 0.01 g	2.07 ± 0.07 f
Laoxihu	8.39	11.93 ± 0.50 d	25.97 ± 0.15 c	164.67 ± 0.67 g	0.58 ± 0.00 d	2.53 ± 0.03 e
Meicun	7.53	15.43 ± 0.12 c	50.60 ± 0.20 a	132.33 ± 0.67 h	0.82 ± 0.00 c	2.43 ± 0.03 e
Sanbao	7.50	10.40 ± 0.20 e	21.40 ± 0.36 e	222.00 ± 0.58 d	0.53 ± 0.01 e	4.43 ± 0.03 b
Lianfeng	8.14	15.43 ± 0.37 c	16.10 ± 0.10 g	254.33 ± 0.33 b	1.00 ± 0.01 a	5.93 ± 0.09 a

Values are given as the mean or mean ± standard deviation (*n* = 3), and the different letters within each column were significantly different (*p* < 0.05).

### 3.2. Regional Variation in Hami Melon Fruit Quality

#### 3.2.1. Morphological Traits

Fruit morphology varied among sites (Table 4). Mean fruit mass ranged from 1.94 ± 0.03 kg at Xiquan to 2.53 ± 0.15 kg at Sanbao. Fruits from Sanbao, Huaxi and Laoxihu were heavier than those from Xiquan and Meicun (*p* < 0.05). Fruit length ranged from 19.37 ± 0.41 to 23.47 ± 0.94 cm and fruit width from 14.27 ± 0.03 to 16.60 ± 0.23 cm. Huaxi fruit were the most elongated (shape index 1.62 ± 0.04), whereas Laoxihu fruit were the least elongated (1.23 ± 0.00). Yuanquan and Lianfeng had the thickest flesh (5.00 ± 0.00 and 5.00 ± 0.06 cm, respectively).

Among the measured traits, the CV values of all morphological parameters—including fruit mass, fruit length, fruit width, fruit shape index, and flesh thickness—were less than 0.1 (Table 4). This low variability indicated that these phenotypic characteristics were genetically relatively stable and less susceptible to environmental modulation under the experimental conditions.

#### 3.2.2. Texture Profile Attributes

Texture profile analysis revealed substantial site-to-site variation in mechanical eating-quality traits (Table 4). TPA hardness ranged from 24.09 ± 0.70 to 43.62 ± 0.40 N and adhesiveness from 0.06 ± 0.01 to 0.16 ± 0.00 mJ. Huaxi fruit had the greatest TPA hardness and adhesiveness, whereas Laoxihu fruit had the lowest values. Dachanghe showed the highest springiness, gumminess and chewiness. By contrast, Sanbao fruit had the lowest gumminess and chewiness, indicating a markedly different texture profile despite its high fruit mass.

Notably, chewiness had a CV greater than 0.3 (high variation), and the CVs of TPA hardness, adhesiveness, springiness and gumminess ranged between 0.1 and 0.3 (moderate variation), while cohesiveness had a CV less than 0.1 (low variation) (Table 4). These results indicated that all texture indices except cohesiveness were susceptible to environmental regulation, with chewiness being the most sensitive to environmental factors.

#### 3.2.3. Soluble Sugars and Organic Acids

Soluble-sugar composition differed strongly among sites (Table 5). Total sugar ranged from 4.94 ± 0.01 g/100 g·FW in Meicun to 13.19 ± 0.08 g/100 g·FW in Sanbao. The fruit sweetness index followed a similar pattern, ranging from 6.04 ± 0.02 to 14.82 ± 0.21%. Sanbao accumulated the most sucrose (8.97 ± 0.01 g/100 g·FW), whereas Meicun accumulated the least (0.32 ± 0.00 g/100 g·FW). Total organic acid ranged from 4.44 ± 0.04 g/kg·FW at Dachanghe to 6.19 ± 0.01 g/kg·FW at Meicun. Meicun had the highest malic acid, while Meicun and Sanbao shared the highest citric acid group.

**Table 4:** Morphological and texture traits of Hami melon fruit from eight production sites.

Site	Fruit Mass (kg)	Fruit Length (cm)	Fruit Width (cm)	Shape Index	Flesh Thickness (cm)	TPA Hardness (N)	Adhesiveness (mJ)	Cohesiveness	Springiness (mm)	Gumminess (N)	Chewiness (mJ)
Xiquan	1.94 ± 0.03 b	19.73 ± 0.18 b	14.27 ± 0.03 b	1.38 ± 0.01 b	4.10 ± 0.23 b	41.26 ± 1.10 b	0.11 ± 0.00 b	0.41 ± 0.01 ab	1.74 ± 0.03 a	17.15 ± 0.29 a	31.01 ± 0.73 ab
Dachanghe	2.18 ± 0.20 ab	20.20 ± 0.29 b	14.97 ± 0.70 b	1.35 ± 0.05 b	4.43 ± 0.22 b	40.52 ± 0.97 b	0.10 ± 0.01 b	0.42 ± 0.01 ab	1.77 ± 0.06 a	18.12 ± 0.62 a	31.94 ± 0.67 a
Huaxi	2.46 ± 0.15 a	23.47 ± 0.94 a	14.47 ± 0.24 b	1.62 ± 0.04 a	4.50 ± 0.20 b	43.62 ± 0.40 a	0.16 ± 0.00 a	0.41 ± 0.01 ab	1.73 ± 0.03 a	17.23 ± 0.47 a	29.67 ± 0.21 b
Yuanquan	2.34 ± 0.06 ab	20.77 ± 0.20 b	14.73 ± 0.13 b	1.41 ± 0.01 b	5.00 ± 0.00 a	35.83 ± 0.18 c	0.12 ± 0.01 b	0.41 ± 0.01 ab	1.66 ± 0.03 ab	14.27 ± 0.35 b	25.79 ± 0.47 c
Laoxihu	2.46 ± 0.07 a	20.35 ± 0.20 b	16.60 ± 0.23 a	1.23 ± 0.00 c	4.45 ± 0.03 b	24.09 ± 0.70 e	0.06 ± 0.01 c	0.43 ± 0.03 a	1.37 ± 0.06 c	12.35 ± 0.35 c	18.22 ± 0.61 e
Meicun	2.01 ± 0.09 b	20.70 ± 0.58 b	14.60 ± 0.44 b	1.42 ± 0.04 b	4.23 ± 0.15 b	34.02 ± 1.00 c	0.10 ± 0.00 b	0.45 ± 0.02 a	1.56 ± 0.01 b	15.10 ± 0.23 b	22.60 ± 0.40 d
Sanbao	2.53 ± 0.15 a	20.60 ± 0.69 b	16.50 ± 0.45 a	1.25 ± 0.02 c	4.23 ± 0.12 b	26.67 ± 0.43 d	0.12 ± 0.00 b	0.40 ± 0.01 ab	1.38 ± 0.01 c	6.66 ± 0.66 d	9.22 ± 0.95 f
Lianfeng	2.30 ± 0.15 ab	19.37 ± 0.41 b	15.30 ± 0.25 b	1.27 ± 0.01 c	5.00 ± 0.06 a	33.85 ± 0.64 c	0.10 ± 0.01 b	0.38 ± 0.01 b	1.69 ± 0.03 a	11.31 ± 0.65 c	19.63 ± 1.18 e
CV	0.09	0.06	0.06	0.09	0.08	0.20	0.24	0.05	0.10	0.27	0.33

Values are given as the mean ± standard deviation (*n* = 3), and the different letters within each column were significantly different (*p* < 0.05).

**Table 5:** Soluble-sugar and organic-acid composition of Hami melon fruit from eight production sites.

Site	Fructose (g/100 g·FW)	Glucose (g/100 g·FW)	Sucrose (g/100 g·FW)	Total Sugar (g/100 g·FW)	Sweetness (%)	Malic Acid (g/kg·FW)	Citric Acid (g/kg·FW)	Total Acid (g/kg·FW)	Sugar–Acid Ratio
Xiquan	4.24 ± 0.05 ab	2.08 ± 0.02 c	3.27 ± 0.04 f	9.60 ± 0.07 d	12.15 ± 0.10 e	2.73 ± 0.00 e	2.23 ± 0.00 c	4.96 ± 0.01 e	19.35 ± 0.13 d
Dachanghe	4.36 ± 0.04 a	2.52 ± 0.03 a	4.80 ± 0.01 b	11.68 ± 0.03 b	14.19 ± 0.06 b	2.70 ± 0.02 e	1.74 ± 0.02 f	4.44 ± 0.04 f	26.28 ± 0.24 a
Huaxi	4.03 ± 0.17 b	2.55 ± 0.04 a	2.59 ± 0.01 g	9.17 ± 0.14 e	11.43 ± 0.28 f	3.29 ± 0.03 b	1.82 ± 0.01 e	5.11 ± 0.02 d	17.95 ± 0.20 e
Yuanquan	3.68 ± 0.03 c	2.58 ± 0.03 a	4.59 ± 0.04 c	10.84 ± 0.02 c	12.83 ± 0.04 d	3.12 ± 0.00 c	2.34 ± 0.01 b	5.46 ± 0.01 c	19.84 ± 0.01 d
Laoxihu	3.18 ± 0.05 d	1.06 ± 0.04 e	4.49 ± 0.04 d	8.73 ± 0.09 f	10.80 ± 0.12 g	2.99 ± 0.00 d	2.12 ± 0.00 d	5.11 ± 0.01 d	17.10 ± 0.17 f
Meicun	2.37 ± 0.01 f	2.25 ± 0.00 b	0.32 ± 0.00 h	4.94 ± 0.01 g	6.04 ± 0.02 h	3.57 ± 0.00 a	2.62 ± 0.01 a	6.19 ± 0.01 a	7.99 ± 0.00 g
Sanbao	2.76 ± 0.15 e	1.47 ± 0.06 d	8.97 ± 0.01 a	13.19 ± 0.08 a	14.82 ± 0.21 a	2.98 ± 0.01 d	2.64 ± 0.03 a	5.62 ± 0.02 b	23.50 ± 0.17 b
Lianfeng	4.49 ± 0.07 a	2.31 ± 0.07 b	3.89 ± 0.02 e	10.69 ± 0.12 c	13.36 ± 0.15 c	2.66 ± 0.00 f	2.30 ± 0.02 b	4.96 ± 0.02 e	21.57 ± 0.28 c
CV	0.22	0.26	0.59	0.25	0.23	0.11	0.15	0.10	0.28

Values are given as the mean ± standard deviation (*n* = 3), and the different letters within each column were significantly different (*p* < 0.05).

The resulting sugar–acid ratio ranged more than threefold, from 7.99 ± 0.00 at Meicun to 26.28 ± 0.24 at Dachanghe (Table 5). Thus, the sites with the greatest sugar concentration and the most favorable sugar–acid balance were not identical: Sanbao maximized total sugar, whereas Dachanghe combined high sugar with the lowest total acid.

Fructose, glucose, total sugar, sweetness, malic acid, citric acid, total acid and sugar–acid ratio all exhibited moderate variation (CV 0.1–0.3), while sucrose was the only high-variation trait (CV > 0.3) (Table 5). These results indicated that most soluble-sugar and organic acid-related indices were susceptible to environmental modulation, with sucrose being the most environmentally sensitive.

#### 3.2.4. Mineral Elements

Fruit mineral composition varied markedly among sites (Table 6). Calcium was highest at Lianfeng (53.30 ± 0.38 mg/kg·DW) and lowest at Sanbao (15.35 ± 0.31 mg/kg·DW). Iron peaked at Yuanquan and Lianfeng (3.76 ± 0.02 mg/kg·DW), while magnesium was highest at Huaxi (272.43 ± 1.50 mg/kg·DW). Xiquan had the greatest zinc concentration (1.42 ± 0.02 mg/kg·DW). Potassium, the most abundant measured mineral, ranged from 2027.33 ± 2.28 mg/kg·DW at Meicun to 4085.67 ± 0.43 mg/kg·DW at Sanbao.

Among the measured mineral elements, magnesium and potassium both fell into the moderate-variation category (CV 0.1–0.3) (Table 6). In comparison, iron, calcium, and zinc were categorized as high variation (CV > 0.3) (Table 6). This variability pattern indicated that the tested mineral indices were generally susceptible to environmental influence, and iron was the most environmentally sensitive element among them.

**Table 6:** Mineral element content in Hami melon fruit from eight production sites.

Site	Calcium (mg/kg·DW)	Iron (mg/kg·DW)	Magnesium (mg/kg·DW)	Zinc (mg/kg·DW)	Potassium (mg/kg·DW)
Xiquan	50.03 ± 0.34 b	3.33 ± 0.02 b	214.27 ± 1.92 b	1.42 ± 0.02 a	2713.83 ± 4.70 e
Dachanghe	16.47 ± 0.08 f	2.21 ± 0.01 c	143.13 ± 0.52 g	0.90 ± 0.02 d	3591.20 ± 3.06 b
Huaxi	26.85 ± 0.08 e	2.04 ± 0.05 d	272.43 ± 1.50 a	0.45 ± 0.02 g	2458.23 ± 4.88 g
Yuanquan	47.91 ± 0.19 d	3.76 ± 0.02 a	210.33 ± 0.75 c	1.27 ± 0.01 b	2814.57 ± 3.68 d
Laoxihu	26.75 ± 0.22 e	1.19 ± 0.02 f	131.07 ± 0.20 h	0.71 ± 0.02 e	2913.70 ± 5.16 c
Meicun	48.73 ± 0.17 c	1.56 ± 0.00 e	185.23 ± 0.66 e	0.92 ± 0.02 d	2027.33 ± 2.28 h
Sanbao	15.35 ± 0.31 g	1.00 ± 0.02 g	177.53 ± 0.87 f	0.60 ± 0.01 f	4085.67 ± 0.43 a
Lianfeng	53.30 ± 0.38 a	3.76 ± 0.03 a	200.73 ± 1.46 d	1.03 ± 0.01 c	2487.47 ± 4.12 f
CV	0.45	0.48	0.23	0.36	0.23

Values are given as the mean ± standard deviation (*n* = 3), and the different letters within each column indicate significant differences (*p* < 0.05).

#### 3.2.5. Bioactive Compounds and Antioxidant Capacity

Total polyphenol content was highest in Xiquan fruit (0.93 ± 0.01 mg/g·FW) and lowest in Huaxi and Sanbao fruit (0.78 ± 0.01 mg/g·FW and 0.78 ± 0.02 mg/g·FW) (Table 7). Total flavonoid content ranged from 4.44 ± 0.12 mg/100 g·FW at Sanbao to 8.19 ± 0.05 mg/100 g·FW at Dachanghe. Xiquan also showed the highest DPPH (73.91 ± 2.51%) and ABTS (77.08 ± 2.08%) radical scavenging capacities, whereas Huaxi had the lowest values for both assays. Ascorbic acid and protein both reached their highest levels at Sanbao (14.20 ± 0.06 mg/100 g·FW and 0.89 ± 0.00 g/100 g·FW, respectively), whereas Lianfeng had the lowest concentrations of ascorbic acid (11.67 ± 0.03 mg/100 g·FW) and protein (0.64 ± 0.00 g/100 g·FW) (Table 7).

Across all tested samples, total polyphenols, ABTS scavenging capacity, and ascorbic acid showed low variability (CV < 0.1), while total flavonoids, DPPH scavenging capacity, and protein exhibited moderate variability (CV 0.1–0.3) (Table 7). This pattern indicates that the latter three traits were more susceptible to environmental modulation.

**Table 7:** Bioactive compounds and antioxidant-related traits of Hami melon fruit from eight production sites.

Site	Total Polyphenols (mg/g·FW)	Total Flavonoids (mg/100 g·FW)	DPPH Scavenging Capacity (%)	ABTS Scavenging Capacity (%)	Ascorbic Acid (mg/100 g·FW)	Protein (g/100 g·FW)
Xiquan	0.93 ± 0.01 a	5.56 ± 0.08 d	73.91 ± 2.51 a	77.08 ± 2.08 a	12.53 ± 0.07 b	0.75 ± 0.00 e
Dachanghe	0.83 ± 0.02 b	8.19 ± 0.05 a	65.22 ± 2.51 c	68.75 ± 0.00 bc	12.00 ± 0.06 e	0.66 ± 0.00 g
Huaxi	0.78 ± 0.01 c	7.52 ± 0.12 b	55.07 ± 1.45 d	56.25 ± 0.00 d	12.20 ± 0.06 cd	0.79 ± 0.00 d
Yuanquan	0.83 ± 0.01 b	7.20 ± 0.03 c	66.67 ± 1.45 bc	72.92 ± 2.08 ab	12.23 ± 0.09 c	0.86 ± 0.00 b
Laoxihu	0.83 ± 0.01 b	7.31 ± 0.06 bc	72.46 ± 1.45 ab	75.00 ± 0.00 a	12.03 ± 0.03 de	0.72 ± 0.00 f
Meicun	0.84 ± 0.01 b	4.94 ± 0.03 e	55.07 ± 1.45 d	66.67 ± 2.08 c	12.13 ± 0.07 cde	0.82 ± 0.00 c
Sanbao	0.78 ± 0.02 c	4.44 ± 0.12 f	57.97 ± 1.45 d	66.67 ± 2.08 c	14.20 ± 0.06 a	0.89 ± 0.00 a
Lianfeng	0.84 ± 0.01 b	7.42 ± 0.08 bc	69.57 ± 2.51 abc	72.92 ± 2.08 ab	11.67 ± 0.03 f	0.64 ± 0.00 h
CV	0.06	0.21	0.12	0.09	0.06	0.12

Values are given as the mean ± standard deviation (*n* = 3), and the different letters within each column indicate significant differences (*p* < 0.05).

### 3.3. Multivariate Quality Assessment

#### 3.3.1. Principal Component Analysis

Exploratory PCA reduced the 31 quality variables to five components with eigenvalues > 1 (Table 8). PC1-PC5 explained 31.760%, 25.498%, 19.479%, 10.371% and 7.633% of total variance, respectively, for a cumulative 94.741%. PC1 was primarily associated with fruit mass, fruit width, fruit shape index, TPA hardness, springiness, gumminess, chewiness, fructose, glucose, sucrose, citric acid, calcium, magnesium, iron, potassium, and ascorbic acid. PC2 showed strong correlations with fruit length, fruit shape index, cohesiveness, fructose, sucrose, total sugar, sweetness, malic acid, total acid, sugar–acid ratio, potassium, free radical scavenging capacity (DPPH and ABTS), and protein. PC3 was closely linked to fruit mass, fruit length, fruit shape index, adhesiveness, calcium, zinc, total polyphenols, and the free radical scavenging capacity of DPPH and ABTS. PC4 was associated mainly with cohesiveness, citric acid, magnesium, and total flavonoids, whereas PC5 was dominated by flesh thickness.

#### 3.3.2. Comprehensive Evaluation of Fruit Quality

Variance-weighted component scores yielded a composite quality index for each site (Table 9). Dachanghe ranked first (47.36), followed by Xiquan (41.07) and Huaxi (32.53). Lianfeng and Yuanquan occupied intermediate positions. Sanbao ranked sixth because its high sugar and potassium concentrations were offset by the low texture and poor performance of several mineral and phenolic traits. Laoxihu and Meicun had the two lowest composite scores.

#### 3.3.3. Cluster Analysis of Fruit Quality

**Figure 1:**
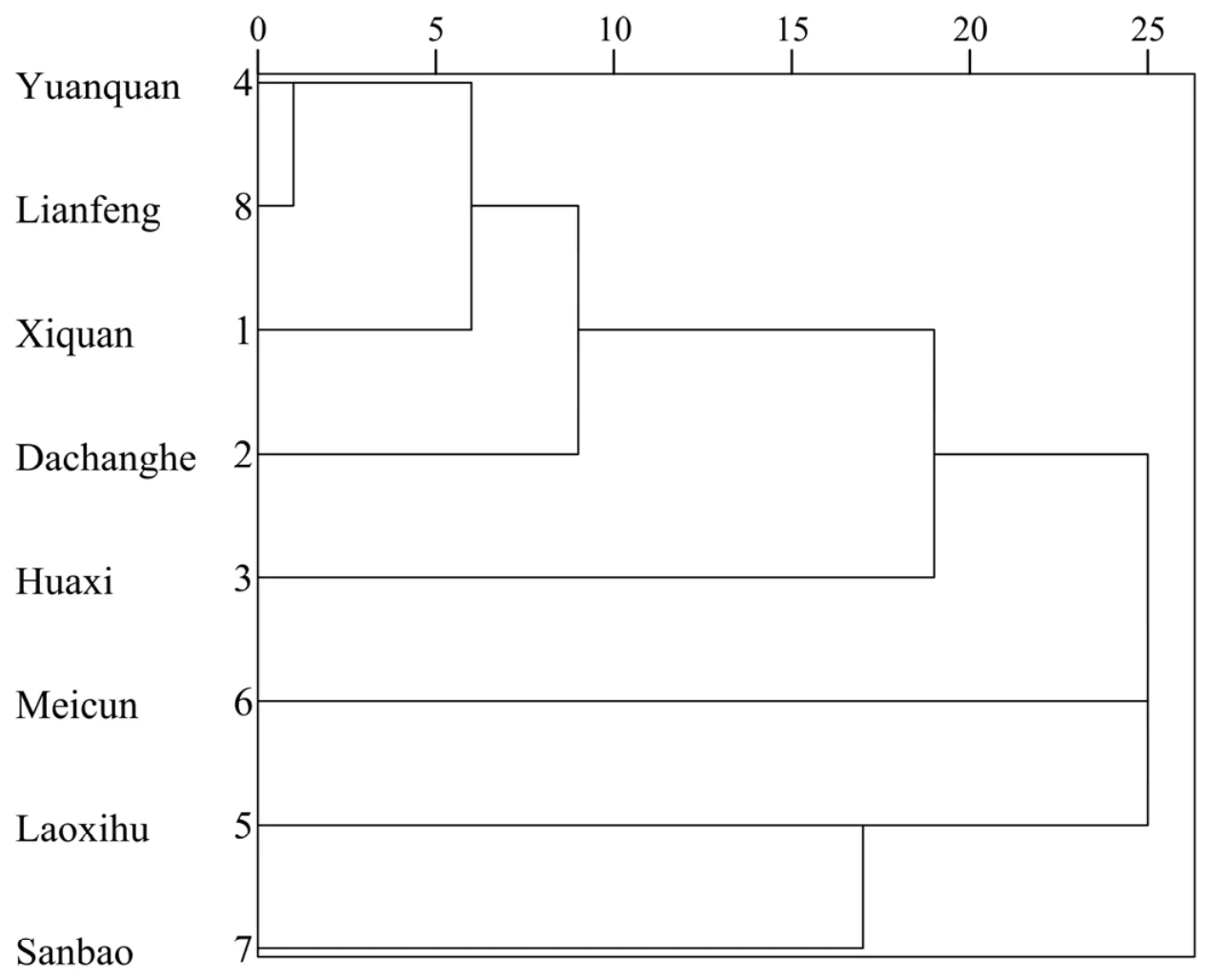
Cluster analysis based on 31 quality traits.

Cluster analysis divided Hami melons from eight regions into three categories (Figure 1). Yuanquan, Lianfeng, Xiquan, Dachanghe and Huaxi formed the first category; Laoxihu and Sanbao formed the second category, while Meicun formed a separate third category. The classification results were consistent with the F values obtained from the comprehensive evaluation.

#### 3.3.4. Construction of Fruit Quality Evaluation Model

Stepwise regression analysis was performed using all 31 measured traits of Hami melon fruits as candidate predictors, with the comprehensive quality score (F) designated as the response variable. This procedure yielded the descriptive equation F = −285.671 + 25.253 × fructose + 3.933 × TPA hardness + 5.703 × total sugar (adjusted R^2^ = 0.981, nominal *p* < 0.001; Table 10). The results of the multiple linear regression indicated that the fruit quality of Hami melon was significantly influenced by fructose, TPA hardness, and total sugar. Fructose alone accounted for 78.9% of the variation in quality traits, demonstrating a high degree of explanatory power. Collectively, fructose, TPA hardness, and total sugar explained 98.1% of the fruit quality variation, indicating that these three indicators could effectively capture the overall quality of Hami melon fruit. Therefore, when evaluating the fruit quality of Hami melons from different producing areas, priority can be given to measuring these three indicators for an effective quality assessment.

### 3.4. Associations Among Quality Traits and Environmental Variables

#### 3.4.1. Correlation Analysis Between Fruit Quality Traits

**Figure 2:**
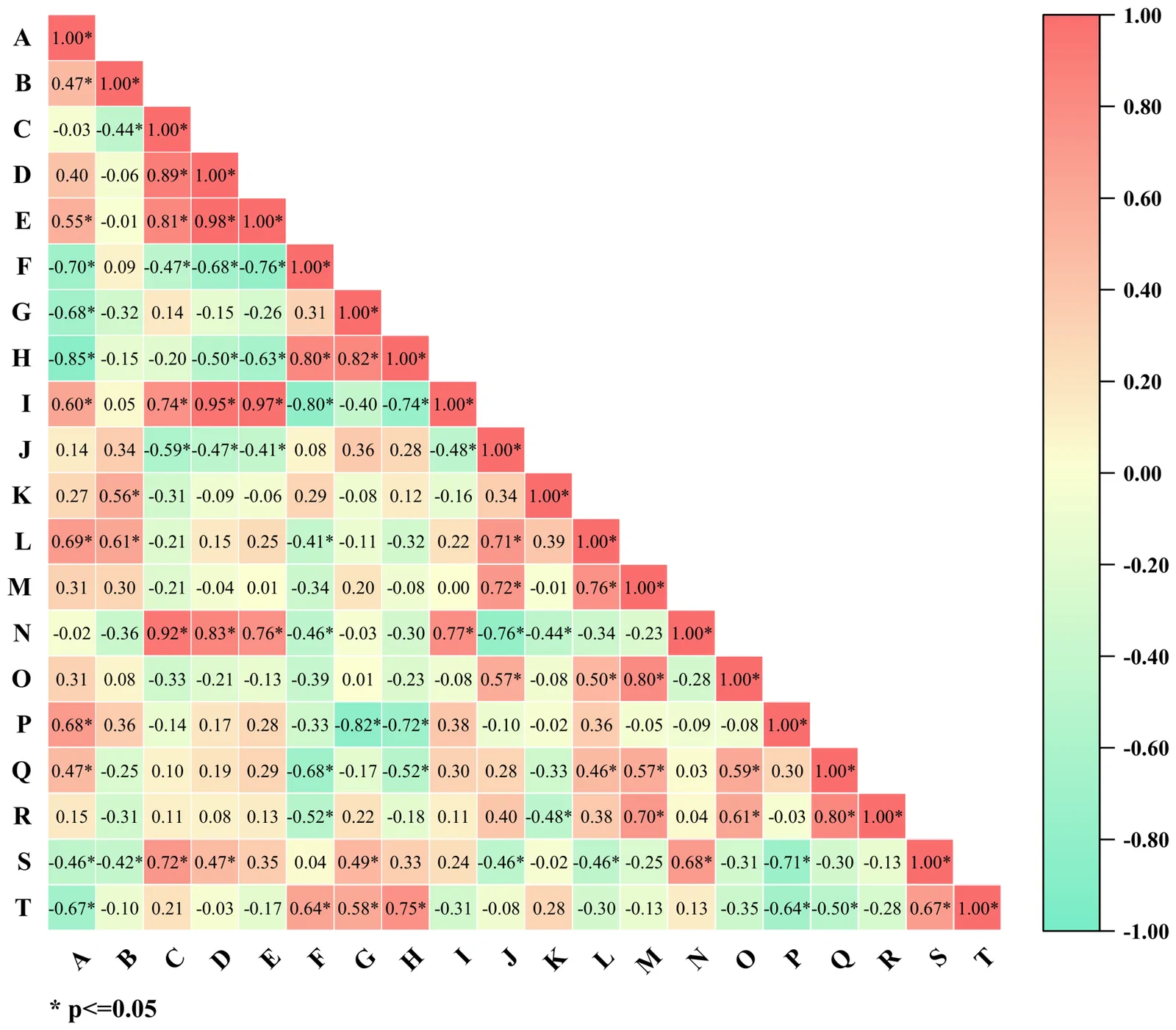
Pearson correlations among 20 fruit quality traits. A, fructose; B, glucose; C, sucrose; D, total sugar; E, sweetness index; F, malic acid; G, citric acid; H, total acid; I, sugar–acid ratio; J, calcium; K, magnesium; L, iron; M, zinc; N, potassium; O, total polyphenols; P, total flavonoids; Q, DPPH radical scavenging capacity; R, ABTS radical scavenging capacity; S, ascorbic acid; T, protein. * Nominal *p* ≤ 0.05.

Figure 2 presents the pairwise correlations among fruit quality attributes. Calcium exhibited strong negative correlations with sucrose (r = −0.59), total sugar (r = −0.47), sweetness (r = −0.41), sugar–acid ratio (r = −0.48), potassium (r = −0.76) and ascorbic acid (r = −0.46), while showing significant positive associations with iron (r = 0.71), zinc (r = 0.72) and total polyphenols (r = 0.57). Magnesium correlated positively with glucose (r = 0.56), and negatively with potassium (r = −0.44) and ABTS radical scavenging capacity (r = −0.48). Iron had significant positive links to fructose (r = 0.69), glucose (r = 0.66), calcium (r = 0.71), zinc (r = 0.76), total polyphenols (r = 0.50), and DPPH free radical scavenging capacity (r = 0.46), alongside negative correlations with malic acid (r = −0.41) and ascorbic acid (r = −0.46). Zinc showed strong positive correlations with total polyphenols (r = 0.80), DPPH (r = 0.57) and ABTS (r = 0.80) scavenging capacities. Potassium was significantly positively correlated with sucrose (r = 0.92), total sugar (r = 0.83), sweetness (r = 0.76), sugar–acid ratio (r = 0.77) and ascorbic acid (r = 0.68), and negatively correlated with malic acid (r = −0.46).

Additionally, total polyphenols correlated positively with both DPPH (r = 0.59) and ABTS (r = 0.61) activities. Total flavonoids showed negative associations with ascorbic acid (r = −0.71) and protein (r = −0.64). DPPH activity was positively linked to ABTS activity (r = 0.80) and negatively linked to protein content (r = −0.50). Ascorbic acid exhibited a significant positive correlation with protein (r = 0.67).

#### 3.4.2. Correlation Analysis Between Fruit Quality and Climatic Variables

**Figure 3:**
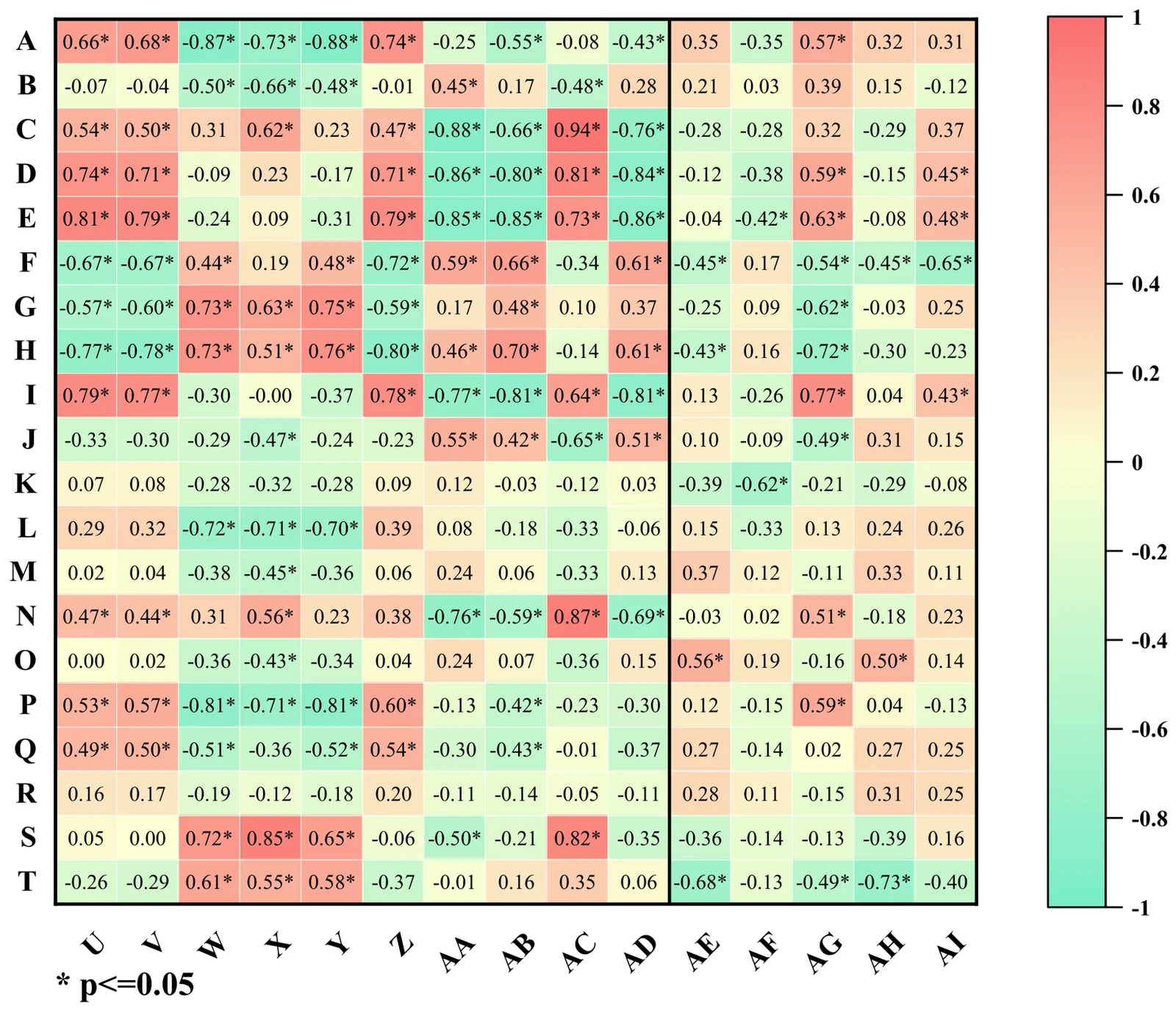
Pearson correlations between 20 fruit quality traits and 15 climatic or soil variables. A, fructose; B, glucose; C, sucrose; D, total sugar; E, sweetness index; F, malic acid; G, citric acid; H, total acid; I, sugar–acid ratio; J, calcium; K, magnesium; L, iron; M, zinc; N, potassium; O, total polyphenols; P, total flavonoids; Q, DPPH radical scavenging capacity; R, ABTS radical scavenging capacity; S, ascorbic acid; T, protein; U, sunshine duration; V, solar radiation; W, mean temperature; X, mean maximum temperature; Y, mean minimum temperature; Z, diurnal temperature range; AA, rainy days; AB, precipitation; AC, evaporation; AD, relative humidity; AE, soil organic matter; AF, soil available phosphorus; AG, soil available potassium; AH, soil total nitrogen; AI, soil cation exchange capacity. * Nominal *p* ≤ 0.05.

Figure 3 illustrates the correlations between fruit quality and climatic variables. Fructose, sucrose, total sugar, sweetness, sugar–acid ratio, potassium, total flavonoids, and DPPH radical scavenging capacity in Hami melon fruits showed strong positive correlations with light-related parameters (mean monthly sunshine duration and solar radiation), while malic acid, citric acid, and total acid exhibited significant negative correlations with these indicators. Sucrose, total sugar, sweetness, sugar–acid ratio, and potassium were markedly inversely associated with humidity metrics (mean monthly rainy days, total precipitation, and relative humidity), whereas malic acid and total acid were positively correlated with these humidity factors.

Furthermore, fructose, glucose, iron and total flavonoids in fruits had significant negative correlations with temperature-related indices (monthly mean, maximum and minimum temperature). By contrast, citric acid, total acid, ascorbic acid and protein showed significant positive associations with the above temperature parameters. Notably, multiple key quality attributes of Hami melon—fructose, sucrose, total sugar, sweetness, sugar–acid ratio, total flavonoids and DPPH scavenging capacity—were all significantly positively correlated with monthly mean diurnal temperature range, with r values of 0.74, 0.47, 0.71, 0.79, 0.78, 0.60 and 0.54, respectively. Conversely, malic acid, citric acid and total acid were significantly negatively correlated with this diurnal temperature variable, with r values of −0.72, −0.59 and −0.80, respectively.

#### 3.4.3. Correlation Analysis Between Fruit Quality and Soil Properties

Figure 3 presents the significant correlations between Hami melon fruit quality and soil properties. Fruit total polyphenol content was positively correlated with soil organic matter (r = 0.56), while malic acid, total acid and protein showed negative correlations with this soil parameter (r = −0.45, −0.43 and −0.68, respectively). Fruit sweetness and magnesium were significantly inversely associated with soil available phosphorus (r = −0.42 and −0.62, respectively).

Fructose, total sugar, sweetness, sugar–acid ratio, potassium and total flavonoids exhibited significant positive correlations with soil available potassium (r = 0.57, 0.59, 0.63, 0.77, 0.51 and 0.59, respectively), whereas malic acid, citric acid, total acid, calcium and protein were negatively correlated with this index (r = −0.54, −0.62, −0.72, −0.49 and −0.49, respectively). Total soil nitrogen showed negative links to fruit malic acid (r = −0.45) and protein (r = −0.73), and a positive link to total polyphenols (r = 0.50). Soil cation exchange capacity was positively correlated with fruit total sugar, sweetness and sugar–acid ratio (r = 0.45, 0.48 and 0.43, respectively), and negatively correlated with malic acid (r = −0.65). These findings identify candidate soil variables for controlled fertilization trials.

## 4. Discussion

Rigorous scientific methods are indispensable for comprehensive evaluation of fruit quality. Widely adopted approaches include the fuzzy mathematics membership function method [11,26,27], gray relational analysis [28,29,30,31], and principal component analysis (PCA) [32,33,34,35]. PCA is widely recognized as a feasible and reliable tool for this purpose [36,37]. It condenses a large dataset of fruit quality traits into a small number of independent principal components and clarifies the relative contribution of each evaluation indicator. On this basis, an objective comprehensive score can be calculated to grade fruit quality, and it has been widely adopted for comprehensive quality evaluation in diverse horticultural crops. For three woody species including *Idesia polycarpa* Maxim., *Chionanthus retusus* and Wangmo *Castanea mollissima*, PCA had served as a robust tool for systematic elite germplasm screening [38,39,40]. A PCA-based predictive model targeting seed traits had also been established for *Prinsepia utilis* to support large-scale germplasm characterization [41]. In *Phyllanthus emblica* L. research, PCA reduced 27 redundant original quality indicators to 12 core traits, significantly streamlining germplasm evaluation workflows [42]. For berry crops, PCA had been applied to screen core quality attributes and constructed predictive evaluation models for strawberry [43], while an independent PCA-driven framework had also been developed to support standardized quality scoring and grading across diverse berry germplasm resources [44]. Notably, most earlier studies assessed quality traits across diverse genotypes within a single environment. However, the present study applied this analytical framework to a single commercial cultivar (‘Xizhoumi 17’) grown in eight geographically distinct producing areas. This design explicitly shifts the research focus from genetic to environmental sources of quality variation, providing an effective complementary perspective to previous germplasm-centered evaluations.

Screening out a compact set of discriminative core quality indicators to enable rapid, low-cost quality prediction carries substantial practical significance for standardized quality management and high-throughput germplasm screening across horticultural and grain crops. A systematic texture quality assessment identified chewiness, adhesiveness and resilience as the most critical texture indicators of muskmelon [10]. Similarly, a dedicated quality evaluation of the ‘Yangjiaocui’ melon cultivar pinpointed a panel of representative indicators, namely fruit longitudinal diameter, flesh thickness rate, hardness, total sugar content and titratable acid content [15]. Combined correlation and PCA analyses identified four core indicators (sucrose pentasaccharide, water content, fructose content and ash content) that fully characterize the overall quality profile of *Helianthus tuberosus* [45], as well as five core indicators (fruit weight, b* value, chewiness, umami intensity, and the W1 W electronic nose sensor response value) for jujube (*Ziziphus jujuba* Mill.) [46]. Using principal component scores derived from multi-trait evaluation, the solid–acid ratio, fruit shape index and fruit hardness were selected as the minimal core evaluation indicators for ‘Fuji’ apples sourced from different producing regions [47]. Finally, via stepwise regression analysis, brown rice quality, chalky grain rate and chalkiness were verified as valid core indicators for accurate brown rice rate prediction [48], while the sugar–acid ratio, fruit weight, fruit shape index, malic acid, citric acid, L-ascorbic acid, quinic acid and total soluble sugar were identified as the key trait set that enables rapid, high-efficiency quality assessment for kiwifruit (*Actinidia eriantha*) [32]. In this work, PCA combined with stepwise regression showed that fructose content, TPA hardness, and total sugar content were the key determinants of Hami melon quality and could be used for its rapid quality evaluation. This finding agrees with the general conclusion of the above studies that sugar-related and textural traits dominate quality variation. In contrast to previous melon studies that emphasized morphological descriptors such as fruit longitudinal diameter and flesh thickness [15], the key indicators in this study were compositional and textural, which suggests that the core indicator set is not fixed, but depends on the dominant source of variation in the population being evaluated.

With respect to environmental regulation, previous studies had systematically confirmed that fruit quality was significantly correlated with climatic conditions [38,45]. The well-established source–sink physiological theory holds that: high radiation promotes carbon assimilation, a large diurnal temperature range boosts higher net carbon gain, while humid or rainy conditions can dilute soluble solid content or disrupt canopy photosynthesis [49]. Consistent with this paradigm, the climate-quality association showed that the sweetness-related indicators of Hami melon fruits in the present study were significantly and positively correlated with light-related parameters and the average diurnal temperature range across the entire fruit development period, while they were negatively correlated with air humidity-related indices. It should be noted, however, that the meteorological data used in this study were obtained from regional meteorological stations rather than from in situ measurements at each sampling site, and the experimental Hami melon plants were grown in a plastic tunnel. The assignment of such station-based data to individual sampling sites inevitably introduces a certain degree of spatial mismatch. This limitation should be taken into account when interpreting the environmental–quality relationships reported here. Nevertheless, the results remain reasonably robust. The observed correlations are not only statistically significant, but also biologically plausible, and they agree well with the source–sink mechanism documented in previous studies [49]. These findings indicated that environments characterized by relatively higher solar radiation, larger diurnal temperature ranges, and lower air humidity were more conducive to the formation of high-quality Hami melon fruits.

Soil nutrients were widely recognized as core regulators of fruit quality formation in horticultural crops [44,50]. Existing field studies had confirmed a tight linkage between edaphic properties and fruit quality across multiple fruit species [51,52]. Correlation analysis revealed that fruit total sugar content, sweetness, and the sugar–acid ratio in Hami melon all exhibited significant positive correlations with soil available potassium content and soil cation exchange capacity, while fruit sweetness and fruit magnesium content showed a significant negative correlation with soil available phosphorus. The positive association between sweetness-related indices and potassium is consistent with the well-established role of K in sugar translocation [53,54] and consistent with the reported watermelon results [51], indicating a conserved mechanism across Cucurbitaceae crops. In contrast, the negative relationship between fruit sweetness and soil available phosphorus observed here differs from the positive correlation between titratable acid and soil available phosphorus reported for citrus [52], implying that the effect of phosphorus on quality is species-specific and possibly related to differences in acid metabolism. Taken together, these cross-species consistencies and differences confirm that the pronounced regional correlation between edaphic properties and fruit quality traits is biologically plausible rather than a random statistical association.

Furthermore, the comprehensive scores revealed a clear regional gradient. Dachanghe, Xiquan and Huaxi from Yumen ranked among the top three, while sites in other agro-climatic zones ranked lower. This identifies Yumen as the optimal producing region for high-quality Hami melon among all tested areas. This ranking is not coincidental, but is closely associated with Yumen’s distinctive agro-ecological conditions, which are characterized by intense solar radiation, low air humidity, and a large diurnal temperature range. Even so, minor differences in soil texture, groundwater depth and local microclimate among Dachanghe, Xiquan and Huaxi still led to small score variations, suggesting that fine-scale optimization of planting site selection could further improve Hami melon quality.

Overall, the present study demonstrated the optimal producing region of Hami melon, and identified fructose content, TPA hardness, and total sugar content of the pulp as the key indexes influencing the overall fruit quality of Hami melon. Furthermore, correlation analysis indicated that appropriately increasing the input of nitrogen fertilizer and organic fertilizer, while reasonably regulating phosphorus fertilizer application, may serve as an effective agronomic practice for improving the quality of Hami melon. However, its scope is limited to one cultivar, one sampling year and a small number of sites, without sufficient replicate farms to separate regional effects from farm-level variation. Further work should directly monitor field environmental conditions, and include multiple farms and growing seasons. The stability of the composite ranking and the performance of the simplified indicator set should then be assessed against independent quality measurements. Until such validation is available, the results presented here should guide additional sampling and controlled experiments rather than direct application to specific fertilizer prescriptions or definitive regional planting recommendations.

## 5. Conclusions

This 2025 multi-site field survey of Hami melon cv. Xizhoumi 17 clarifies the links between regional growing environments and fruit quality, delivering actionable guidance for targeted cultivation and quality optimization across its core planting areas. Significant site-specific divergence in morphological, textural, and compositional traits was observed across the eight sampled locations. Five principal components accounted for 94.74% of the total variance in 31 measured traits, with Dachanghe, Xiquan, and Huaxi ranking top in comprehensive quality performance. Fructose content, TPA hardness, and total sugar content were identified as streamlined proxies for rapid quality estimation, pending independent on-site validation. Sweetness-related traits were strongly promoted by higher light availability and a larger diurnal temperature range, but suppressed by elevated air humidity. Key soil properties including available potassium, cation exchange capacity, organic matter, and total nitrogen showed consistent positive effects on core flavor and nutritional traits. These findings provide direct references for high-quality planting zoning and precision nutrient management, supporting standardized production and characteristic quality branding for the local Hami melon industry.

## Figures and Tables

**Table 1:** Detailed sampling locations, their corresponding numbers and abbreviations.

Sample No.	Sampling Locations	Coordinates	Short Name
1	Xiquan Village, Huahai Town, Yumen City, Gansu Province, China	40.324° N, 97.731° E	Xiquan
2	Dachanghe Modern Agricultural Demonstration Park, Yumen City, Gansu Province, China	40.218° N, 97.827° E	Dachanghe
3	Huaxi Village, Liuhu Town, Yumen City, Gansu Province, China	40.337° N, 97.658° E	Huaxi
4	Yuanquan Village, Dushanzidongxiangzu Town, Yumen City, Gansu Province, China	40.230° N, 97.702° E	Yuanquan
5	Laoxihu Village, Xihu Town, Guazhou County, Jiuquan City, Gansu Province, China	40.466° N, 95.625° E	Laoxihu
6	Meicun Village, Tianya District, Sanya City, Hainan Province, China	18.319° N, 109.395° E	Meicun
7	Sanbao Village, Gaochang District, Turpan City, Xinjiang Uygur Autonomous Region, China	42.912° N, 90.291° E	Sanbao
8	Lianfeng Village, Wuyuan County, Bayannaoer City, Inner Mongolia Autonomous Region, China	40.537° N, 106.579° E	Lianfeng

**Table 2:** Monthly average climatic conditions from April to July 2025.

Region	Sunshine (h)	Solar Rad. (MJ/m^2^)	Tmean (°C)	Tmax (°C)	Tmin (°C)	DTR (°C)	Rainy Days (d)	Precip. (mm)	Evap. (mm)	RH (%)
Yumen	296.23	359.05	18.70	26.25	14.75	11.50	5.00	6.08	179.30	32.61
Guazhou	295.90	358.79	21.70	29.25	17.75	11.50	3.00	1.58	188.48	27.48
Sanya	150.75	326.39	27.70	29.50	24.25	5.25	13.00	166.58	110.15	94.42
Turpan	277.30	353.12	30.83	36.00	25.75	10.25	0.25	0.03	275.23	19.17
Bayannaoer	276.63	354.73	18.50	26.50	15.00	11.50	5.00	29.17	156.98	43.06

**Table 8:** Principal component loadings and scoring coefficients for 31 fruit quality traits.

Evaluation Indexes	PC1		PC2		PC3		PC4		PC5	
	Load Capacity	Weight	Load Capacity	Weight	Load Capacity	Weight	Load Capacity	Weight	Load Capacity	Weight
Fruit mass	−0.583	−0.059	0.118	0.015	0.599	0.099	−0.107	−0.033	−0.472	−0.199
Fruit length	0.150	0.015	−0.568	−0.072	0.739	0.122	−0.155	−0.048	−0.076	−0.032
Fruit width	−0.912	−0.093	0.252	0.032	−0.027	−0.004	−0.243	−0.076	−0.193	−0.082
Flesh thickness	0.222	0.023	0.336	0.042	0.114	0.019	0.166	0.051	−0.834	−0.353
Shape index	0.658	0.067	−0.533	−0.067	0.501	0.083	0.035	0.011	0.070	0.030
TPA hardness	0.882	0.090	−0.025	−0.003	0.352	0.058	0.106	0.033	0.289	0.122
Adhesiveness	0.355	0.036	−0.338	−0.043	0.752	0.125	0.416	0.130	0.107	0.045
Cohesiveness	−0.049	−0.005	−0.555	−0.070	−0.413	−0.068	−0.534	−0.166	0.287	0.121
Springiness	0.926	0.094	0.209	0.026	0.198	0.033	0.129	0.040	0.116	0.049
Gumminess	0.867	0.088	−0.102	−0.013	0.029	0.005	−0.350	−0.109	0.298	0.126
Chewiness	0.910	0.092	0.053	0.007	0.088	0.015	−0.212	−0.066	0.285	0.121
Fructose	0.648	0.066	0.700	0.089	0.236	0.039	0.004	0.001	−0.058	−0.025
Glucose	0.810	0.082	−0.073	−0.009	0.307	0.051	0.249	0.078	−0.058	−0.025
Sucrose	−0.711	−0.072	0.536	0.068	0.343	0.057	0.228	0.071	0.138	0.058
Total sugar	−0.318	−0.032	0.743	0.094	0.487	0.081	0.285	0.089	0.106	0.045
Sweetness	−0.194	−0.020	0.821	0.104	0.468	0.078	0.241	0.075	0.086	0.036
Malic acid	−0.040	−0.004	−0.968	−0.122	0.059	0.010	−0.046	−0.014	−0.131	−0.055
Citric acid	−0.512	−0.052	−0.351	−0.044	−0.457	−0.076	0.615	0.191	−0.072	−0.030
Total acid	−0.346	−0.035	−0.808	−0.102	−0.251	−0.042	0.358	0.111	−0.124	−0.053
Sugar–acid ratio	−0.114	−0.012	0.855	0.108	0.458	0.076	0.075	0.023	0.162	0.068
Calcium	0.517	0.053	−0.127	−0.016	−0.607	−0.101	0.471	0.147	−0.343	−0.145
Magnesium	0.515	0.052	−0.335	−0.042	0.470	0.078	0.502	0.156	−0.142	−0.060
Iron	0.674	0.068	0.430	0.054	−0.160	−0.026	0.492	0.153	−0.276	−0.117
Zinc	0.458	0.047	0.313	0.040	−0.628	−0.104	0.449	0.140	0.152	0.064
Potassium	−0.620	−0.063	0.508	0.064	0.365	0.061	0.019	0.006	0.414	0.175
Total polyphenols	0.478	0.049	0.287	0.036	−0.709	−0.117	0.151	0.047	0.350	0.148
Total flavonoids	0.473	0.048	0.468	0.059	0.280	0.046	−0.518	−0.161	−0.411	−0.174
DPPH scavenging capacity	0.138	0.014	0.734	0.093	−0.538	−0.089	−0.062	−0.019	−0.04	−0.017
ABTS scavenging capacity	−0.051	−0.005	0.549	0.069	−0.791	−0.131	0.119	0.037	0.027	0.011
Ascorbic acid	−0.709	−0.072	−0.079	−0.010	0.281	0.046	0.448	0.139	0.453	0.192
Protein	−0.392	−0.040	−0.598	−0.076	0.186	0.031	0.492	0.153	0.154	0.065
Eigenvalues	9.846		7.904		6.039		3.215		2.366	
Variance contribution (%)	31.760		25.498		19.479		10.371		7.633	
Cumulative variance contribution (%)	94.741									

**Table 9:** Principal component scores and comprehensive quality scores (F) and site rankings.

Site	PC1	PC2	PC3	PC4	PC5	F	Rank
Xiquan	0.87	0.46	−0.90	0.78	1.46	41.07	2
Dachanghe	0.50	1.03	0.52	−1.20	1.00	47.36	1
Huaxi	0.82	−0.91	1.85	−0.35	−0.32	32.53	3
Yuanquan	0.37	0.09	0.01	0.98	−0.77	18.53	5
Laoxihu	−1.00	0.23	−0.81	−1.62	−0.59	−62.97	7
Meicun	0.13	−1.95	−1.07	−0.12	0.13	−66.80	8
Sanbao	−2.02	0.04	0.76	0.96	0.65	−33.36	6
Lianfeng	0.34	1.02	−0.36	0.56	−1.54	23.63	4

**Table 10:** Stepwise regression of composite quality score on measured fruit traits.

Model	Equation	Adjust R^2^	F	*p* Value
1	F = −195.977 + 53.864 × fructose	0.789	27.17	0.002
2	F = −239.454 + 38.141 × fructose + 2.878 × TPA hardness	0.901	32.917	0.001
3	F = −285.671 + 25.253 × fructose + 3.933 × TPA hardness + 5.703 × total sugar	0.981	119.181	<0.001

## Data Availability

The original contributions presented in this study are included in the article. Further inquiries can be directed to the corresponding author.
